# Reducing the Cognitive Footprint of Brain Tumor Surgery

**DOI:** 10.3389/fneur.2021.711646

**Published:** 2021-08-16

**Authors:** Nicholas B. Dadario, Bledi Brahimaj, Jacky Yeung, Michael E. Sughrue

**Affiliations:** ^1^Robert Wood Johnson School of Medicine, Rutgers University, New Brunswick, NJ, United States; ^2^Department of Neurosurgery, Rush University Medical Center, Chicago, IL, United States; ^3^Centre for Minimally Invasive Neurosurgery, Prince of Wales Private Hospital, Sydney, NSW, Australia

**Keywords:** neurosurgery, brain tumor, connectome, cognition, neuroimaging, machine learning, neurorehabilitation

## Abstract

The surgical management of brain tumors is based on the principle that the extent of resection improves patient outcomes. Traditionally, neurosurgeons have considered that lesions in “non-eloquent” cerebrum can be more aggressively surgically managed compared to lesions in “eloquent” regions with more known functional relevance. Furthermore, advancements in multimodal imaging technologies have improved our ability to extend the rate of resection while minimizing the risk of inducing new neurologic deficits, together referred to as the “onco-functional balance.” However, despite the common utilization of invasive techniques such as cortical mapping to identify eloquent tissue responsible for language and motor functions, glioma patients continue to present post-operatively with poor cognitive morbidity in higher-order functions. Such observations are likely related to the difficulty in interpreting the highly-dimensional information these technologies present to us regarding cognition in addition to our classically poor understanding of the functional and structural neuroanatomy underlying complex higher-order cognitive functions. Furthermore, reduction of the brain into isolated cortical regions without consideration of the complex, interacting brain networks which these regions function within to subserve higher-order cognition inherently prevents our successful navigation of true eloquent and non-eloquent cerebrum. Fortunately, recent large-scale movements in the neuroscience community, such as the Human Connectome Project (HCP), have provided updated neural data detailing the many intricate macroscopic connections between cortical regions which integrate and process the information underlying complex human behavior within a brain “connectome.” Connectomic data can provide us better maps on how to understand convoluted cortical and subcortical relationships between tumor and human cerebrum such that neurosurgeons can begin to make more informed decisions during surgery to maximize the onco-functional balance. However, connectome-based neurosurgery and related applications for neurorehabilitation are relatively nascent and require further work moving forward to optimize our ability to add highly valuable connectomic data to our surgical armamentarium. In this manuscript, we review four concepts with detailed examples which will help us better understand post-operative cognitive outcomes and provide a guide for how to utilize connectomics to reduce cognitive morbidity following cerebral surgery.

## Introduction

Modern glioma surgery has advanced based on the understanding that maximal tumor resection improves overall survival (1). While also considering numerous factors such as patient prognosis, tumor topography relative to “eloquent” or “non-eloquent” cerebrum has generally guided the aggressiveness of surgical cytoreduction in hopes of minimizing the risk of inducing new neurologic deficits (2). Such a balance is often referred to as the patient “onco-functional balance.” However, while it is well known that not all cortical tissue is functionally eloquent and the brain is generally resistant to a degree of surgical reduction, glioma patients continue to present post-operatively with poor cognitive functioning limiting social interactions and integration back into the workforce (3–5). If the neurosurgical community is to further consider increasing the extent of resection such as in supramaximal resection, further anatomical and functional information is required to improve our effective navigation of human cerebrum during tumor surgery and to maximize cognitive preservation.

Anatomical familiarity with specific cortical structures and advancements in multimodal imaging have allowed neurosurgeons to minimize surgically induced neurologic deficits related to major functions including language and motor skills. However, such notions are still often unable to explain the subtle deficits seen in patients with higher-order cognitive functions nor can it explain the heterogeneity in cognitive outcomes with lesions located in traditionally “non-eloquent” tissue (3, 5–7). One plausible hypothesis suggests that inter-individual variability in brain network architecture may explain why certain patients cannot safely tolerate resection of tumor in classically non-eloquent tissue that is based on generalized brain atlases (8). As such, awake intraoperative electrical stimulation is often employed on an individual patient basis as a gold standard to identify eloquent cortical regions related language and motor skills and facilitate safe tumor resection (9, 10). However, such methods are still often unable to map more complex cognitive functions, such as cognitive functioning and psychomotor speed, which can involve multiple cortical regions functioning together that are classically less anatomically familiar in the general neurosurgery community. Furthermore, these methods can be invasive, time consuming, and difficult to interpret limiting their widespread adoption and clinical applicability at most centers (11, 12). Similarly, functional and structural neuroimaging data have long provided the medical and scientific community an abundance of highly complex and relevant patient data, but this information too is often highly-dimensional and unable to readily guide clinical decision making (13). Fortunately, recent computational advancements and large scale movements in the neuroscience community have allowed us to take this highly dimensional neuroimaging data and improve previous maps of the human brain in a more digestible framework, offering a unique opportunity to improve neuro-oncological outcomes following cerebral surgery (14–16).

The Human Connectome Project (HCP) recently provided a highly detailed neuroanatomical map of human cerebral cortex allowing a reappraisal of our classical modular maps of the human brain (15). The HCP authors identified 180 unique cortical regions per cerebral hemisphere which are architecturally organized in efficient neural networks within a brain “connectome.” Compared to previous *localizationist* views that suggest isolated cortical regions are dedicated to specialized functions, these networks are functionally and structurally organized in a way that minimizes cost while maximizing information transfer between cortical regions to carry out complex human thinking and behavior. Connectomics in turn provides us an improved understanding of the organization and functional relevance of human cortical and subcortical anatomy (15, 17). As the neurosurgeon begins to enter the new era of connectomic-based surgical targeting, further understanding of the structural and functional connectome provides additional information that can allow neurosurgeons to optimize surgical decision making and extend the rate of resection while minimizing new neurologic deficits related to higher-order cognition, among other functions. Furthermore, additional insight may be gained on the potential for functional reallocation during or after cortical insults and on potential targets in brain networks for modulatory treatments and neurorehabilitation (18, 19). However, the ability for this information to readily guide clinical decision making is still relatively nascent and requires further clarification to optimize its clinical applicability.

In the current paper, we discuss and provide evidence for four concepts which we believe can advance the neurosurgical community toward improving patient morbidity and cognitive functioning following cerebral surgery. Furthermore, we review these promising avenues in light of current neurosurgical practices and the current limitations faced. In order to reduce the cognitive footprint of cerebral surgery on the neurosurgical patient, we discuss the following:

*Concept 1*.*Preserve the core of networks whenever possible*
*Concept 2*.*Consider the full brain ramifications of the action*
*Concept 3*.*Move our thinking toward individual circuits*
*Concept 4*.*Consider the possibility that we can change the brain connectome*

## Preserve the Core of Networks Whenever Possible

### Difficulty in Anatomic Localization and Outcomes in Supratentorial Neurosurgery

Patients with gliomas experience impairments in executive functioning, speed, and memory prior to any treatment and are inherently at increased risks for further neuropsychological decline after surgery (20). As discussed above, despite the improved ability for neurosurgeons to manage neurologic outcomes related to language and some motor skills, patients with glioblastoma (GBM) exhibit executive functioning decline post-operatively that can lower patient quality of life and prevent re-integration into the workforce (21, 22). Even with mapping during awake craniotomies, perhaps due to the lack of monitoring capable of testing the complexity of executive functioning, both psychomotor speed and visuospatial functioning are especially impacted (6). Furthermore, limited by the number of neuropsychological batteries that we currently have and the subsequently limited amount of data reported postoperatively about “non-severe” neurological deficits (i.e., other than aphasia or hemiplegia), it is reasonable to conjecture that declines in neurocognitive functions are vastly underappreciated in this patient population (23).

It is unclear whether anatomical location of the lesion can predict specific neurocognitive deficits. The Glioma Outcome Project showed that functional decline, with the exception of language, is not associated with the dominance of the hemisphere where the tumor is located. This suggests that neurocognitive decline may be based on more complex networks involving both hemispheres as the infiltrative tumor continues to grow (24). Relatedly, there is often conflicting evidence in direct electrical stimulation (DES) treatments due to the arbitrary stimulation paradigm where one cortical region may be stimulated and somehow alter neural activity across multiple, adjacent and long-range brain regions (25). More likely, specific cortical regions act as nodes in a complex neural network system that is connected by distinct white matter pathways in order to transfer information and carry out specialized functions (17). Thus, in order to improve our understanding and treatment of neurologic outcomes, we must contextualize neurosurgery in the setting of brain networks.

### Reimagining the Brain as Networks

To understand how to preserve the core of important networks, we must first understand what brain networks are and how function is organized around them. Brain network organization provides a framework to place different cortical regions within that are strongly *functionally* interconnected between time series (26). Functionally connected regions of a network also tend to be *structurally* connected. This idea is supported by a variety of experimental and computational work and has been replicated by our own team in great detail (14, 17, 27–32). In fact, network analyses partly depend on the observations that the *function* of a neural node is in part determined by its *structural* interconnectedness with other nodes in the network (17). Thus, cortical regions in a network represent the nodes of that network and these nodes are connected by edges referred to as white mattery bundles (17). Together, these relationships constitute the *structural* and *functional connectomes*.

Much of how we understand brain circuitry today originates from research based on graph-based network analysis of resting-state functional MRI (33). Resting-state functional MRI (rsMRI) uses low-frequency fluctuations in blood oxygen level-dependent (BOLD) signals to measure endogenous or spontaneous brain activity (34). Early efforts utilized connectivity-based methods to study networks involved in motor, auditory, visual, language, default-mode, and attention systems (34–38). More recently, graph-theory based approaches have allowed for a model of the brain as a complex meta-network and has allowed for us to quantitatively characterize the organization of all brain regions within different, or shared, individual networks. We will not delve into the technical nuances of graph theoretical analyses, but rather briefly describe seven major networks that comprise our current understanding of the brain connectome in Table 1, including: central executive network (CEN), default mode network (DMN), salience network (SN), sensorimotor network, dorsal attention network (DAN), limbic network, and visual network. By utilizing combined structural-functional information and meta-analytic processing software, our team has been creating anatomically precise cortical maps of these brain networks describing key regions in precise HCP nomenclature and their major cortico-cortical connections (27, 73, 74).

**Table 1:** Seven major brain networks.

**1) Central Executive Network**	**2) Visual Network**
The CEN, in contrast to the DMN, is the external mind that is turned on during active tasks and external thinking involving working memory (39). The CEN works in anticorrelation with the DMN in healthy individuals but works in correlation with the dorsal attention network (DAN) for attention processing, as well as visual spatial planning (40, 41). It comprises regions in the anterior cingulate cortex, the inferior parietal lobe, and the posterior most portions of the middle and inferior temporal gyri (42–44). Aberrations in CEN connectivity with other networks, especially abnormal correlations with DMN, have been implicated in many psychiatric disorders, such as schizophrenia and post-traumatic stress disorder (45, 46).	The visual system is situated mostly in the occipital lobe and is comprised of two major pathways, the dorsal and ventral streams (47). The dorsal stream is involved in the guidance of actions and recognition of objects in space and is connected to the parietal lobe (48). The ventral stream is associated with object recognition and form representation (48). It has strong connections to the medial temporal lobe via the basal, tentorial service (49). Although many separate visual functions in spatial and object discrimination are thought to be housed separately in specific parcellations within this network, these two streams are interconnected via the vertical occipital fasciculus and may participate in more interconnected functions than previously understood (49).
**3) Default Mode Network**	**4) Sensorimotor Network**
The DMN is the internal mind that is at work when an individual is at a resting state, not actively engaged in *externally* oriented tasks or attentional processing. However, during that time, the DMN is not stagnant but its activity increases for internal thought and passive sensory processing (50). It comprises the retrosplenial cortex, inferior parietal cortex, dorsolateral frontal cortex, inferior frontal cortex, left inferior temporal gyrus, and medial frontal regions (51, 52).	The sensorimotor network enables us to assimilate both external and internal stimuli and produce a motor response to these elements. The senses can range from temperature, pressure, and vibration (external) to balance and coordination (internal). It is one of the most studied networks in history, from initial basic understanding of the motor cortex in dogs to the understanding of perceptual changes that occur in conjunction with motor learning (53, 54). Anatomically it involves the primary motor cortex, cingulate cortex, premotor cortex, supplementary motor area, sensory cortices in the parietal lobe (55).
**5) Salience Network**	**6) Limbic Network**
The salience network (SN) serves as an intermediary between the DMN and CEN (56, 57). Independently, the SN is thought to process external stimuli from the outside world and modulates how the different networks view the information (58). The main nodes of the SN are situated in the anterior insula and the dorsal anterior cingulate cortex (58). As the SN is in charge of processing of information from the external world, its hyperactivity can lead to neuroticism or anxiety (heightened sensitivity to outside stimuli) and hypoactivity can be a hallmark of autism (lack of sensitivity to social cues) (19).	The limbic network involves multiple lobes and was initially described to be the central control of emotions (59, 60). Its functions were later found to be much wider in scope, ranging from the memory of olfaction to social recognition (61, 62). The limbic network consists of prefrontal-limbic system, anterior cingulate cortex, medial temporal network, parahippocampal gyrus, olfactory lobe, and the ventral tegmental area (63, 64). Lesions of the limbic system are linked to a variety of psychiatric disorders, including anxiety, bipolar disorder, schizophrenia, and autism (65–68).
	**7) Dorsal Attention Network**
	The dorsal attention network (DAN) is an important circuit that governs attention on an object and goal-directed top-down (knowledge derived from previous experience rather than sensory stimulation) processing (69, 70). It comprises bilaterally the intraparietal sulcus and the frontal eye fields of each hemisphere, which are active when attention is overtly or covertly oriented in space (71). Decreased functional connectivity in DAN has been implicated in increased disease severity of dementia (72).

Each network can be further subdivided based on specialized functions (29, 75, 76). Nonetheless, a few networks which are beneficial to first understand in order to grasp the organizational architecture of neural networks in the human cerebrum and that are especially relevant to neurosurgery in hopes of improving cognitive morbidity can be referred to as the “main cognitive networks” (77). The main cognitive networks refer to the CEN, DMN, and the SN, and provide an axis in which the other networks align (78). The DMN is generally thought to alternate its activity with the CEN in an anticorrelated fashion, in which the DMN activates during passive states of mind while the CEN activates during goal-directed behavior and attentional processing (73). Furthermore, the allocation of resources and switching between these two networks based on stimulus orientation and changes in tasks is thought to be mediated by the SN, a cingulo-opercular network (77). Unsurprisingly, abnormal connectivity or disconnection in these major networks can lead to cognitive depletion and impaired higher-order cognitive abilities, with recent evidence suggesting their dysfunction likely forms the underlying basis of many known neurologic and psychiatric disorders, including schizophrenia, depression, and anxiety (79, 80). Thus, perioperative knowledge of these main cognitive networks is imperative to truly optimize patient cognitive status in cerebral surgery.

### Using Brain Network Maps in Surgery

**Figure 1:**
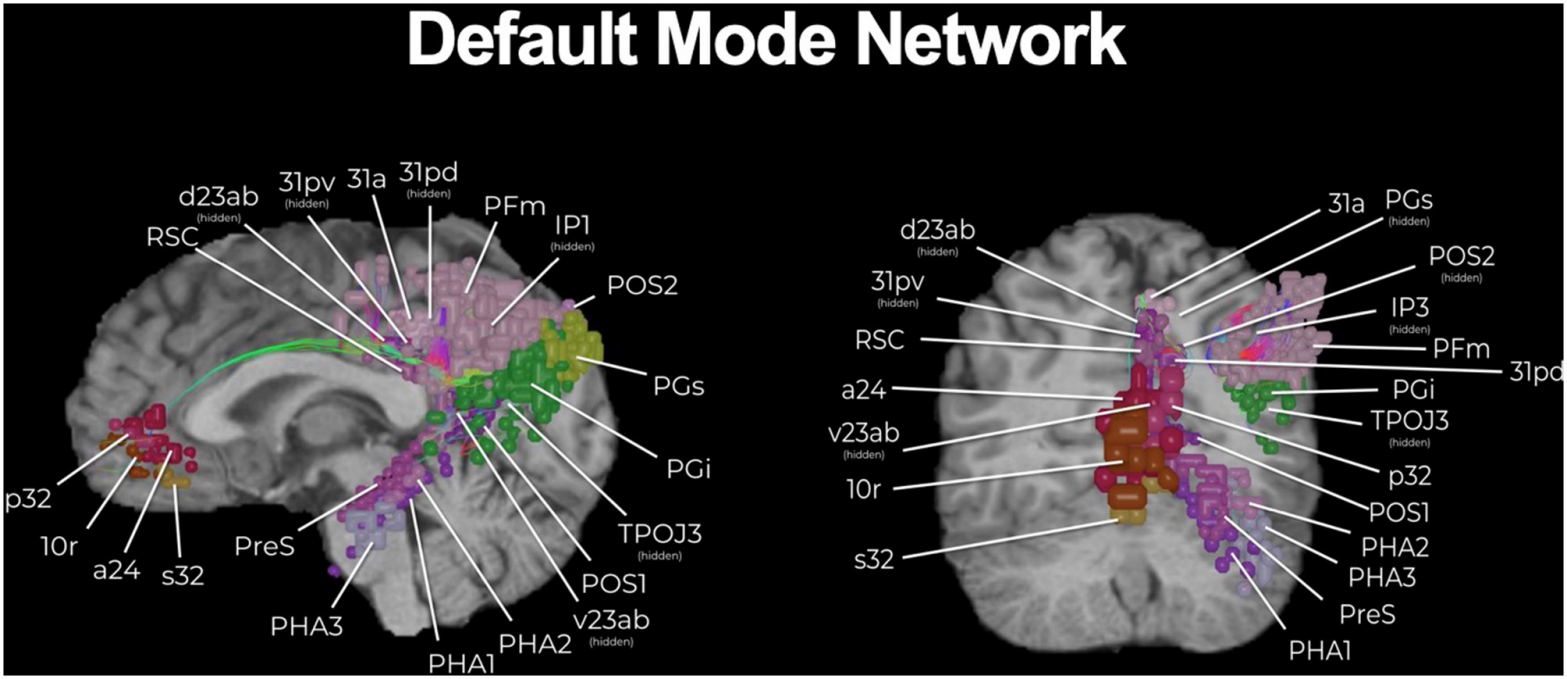
The core of the default mode network (DMN). While a multi-lobar network, the core of the DMN is provided, including its key brain parcellations and connecting structural fibers. Individual regions have been visualized in 3D space and therefore some parcellations have been covered or require multiple views for correct spatial understanding due to parallax. Visualization of tracts have been minimized (thickness and volume) to maximize the visibility of parcellations.

**Figure 2:**
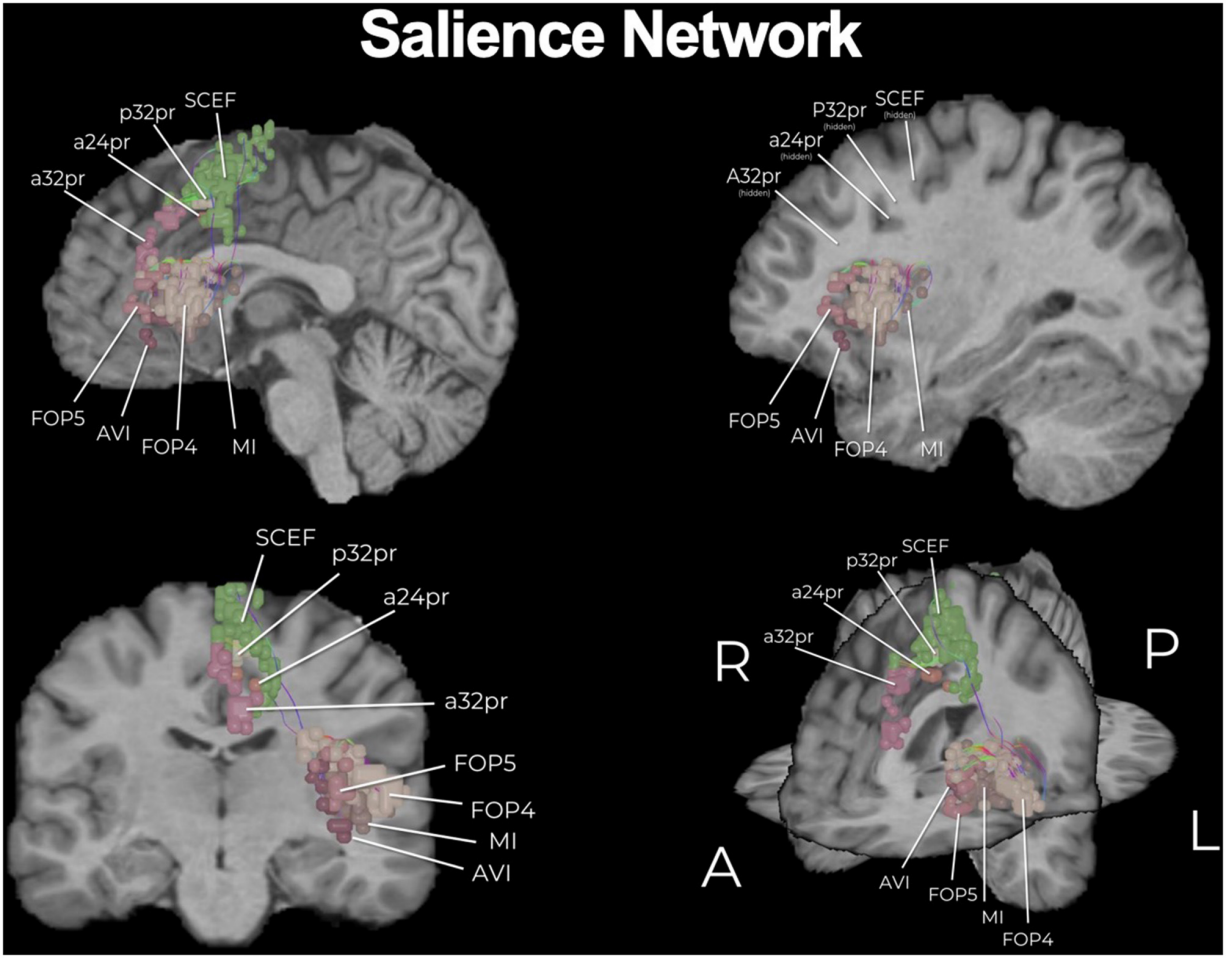
The core of the salience network (SN). Similar to the DMN, the SN is a multi-lobar network and here we show its core. Individual regions have been visualized in 3D space and therefore some parcellations have been covered or require multiple views for correct spatial understanding due to parallax. Visualization of tracts have been minimized (thickness and volume) to maximize the visibility of parcellations.

**Figure 3:**
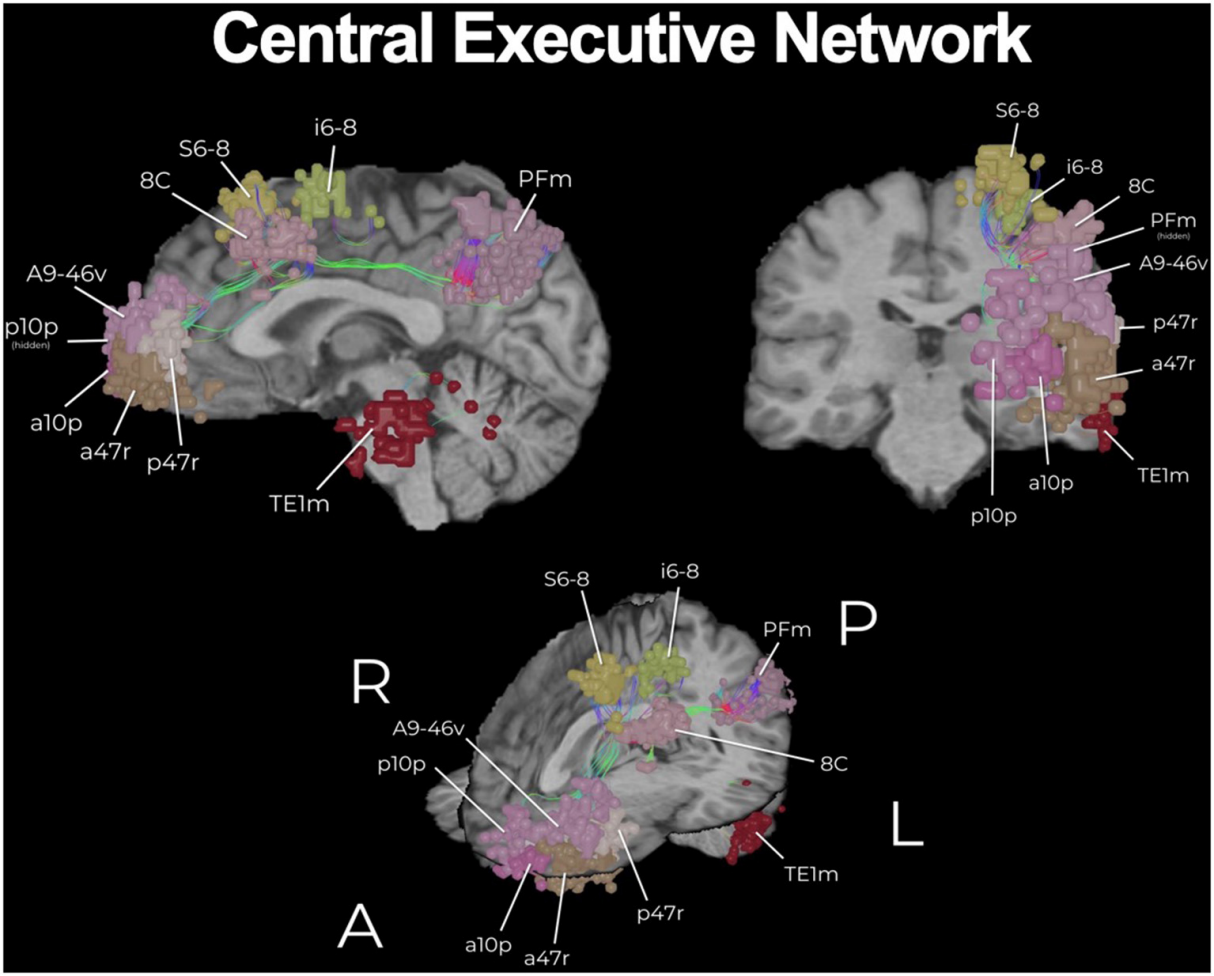
The core of the central executive network (CEN). The CEN is a key multi-lobar control network necessary for effective cognitive functioning and its main core is provided here. Functional regions have been visualized in 3D space and therefore some parcellations have been covered or require multiple views for correct spatial understanding due to parallax. An example of this is the provided with brain parcellation area TE1m (temporal area 1 middle), which is correctly found on the lateral surface of the middle aspect of the middle temporal gyrus (83). Visualization of tracts have been minimized (thickness and volume) to maximize the visibility of parcellations.

The HCP, as well as many others, have provided a plethora of exciting knowledge about the structural and functional connectome (15). However, the clinical translation of such information is still elusive and transforming such information into clinically actionable anatomic information for neurosurgeons has required further work. In addition to describing individual networks, we have previously published a connectomic atlas of the human cerebrum detailing the anatomy and structural and functional connectivity of all 180 precise cortical parcellations according to the HCP authors (81). Brain network maps add an improved understanding of the organization of the human cortex and its underlying subcortical anatomy such that we can make more informed surgical decisions and cause fewer neurologic deficits (14, 82). A number of concepts remain unknown, such as how much of the brain networks must actually be preserved and what fibers can be safely disconnected without compromising network communication and subsequent normal functioning. However, what is generally known suggests that cutting though the cores of the main cognitive networks, consisting of the individual parcellations that are fundamental to the network as well as the interconnections between those main parcellations, causes severe cognitive deficits. To support this concept, we provide a number of important examples below that demonstrate the need for neurosurgeons moving forward to preserve the cores of the main cognitive networks whenever it is surgically feasible. The cores of these structures are visualized in Figures 1–3.

**Figure 4:**
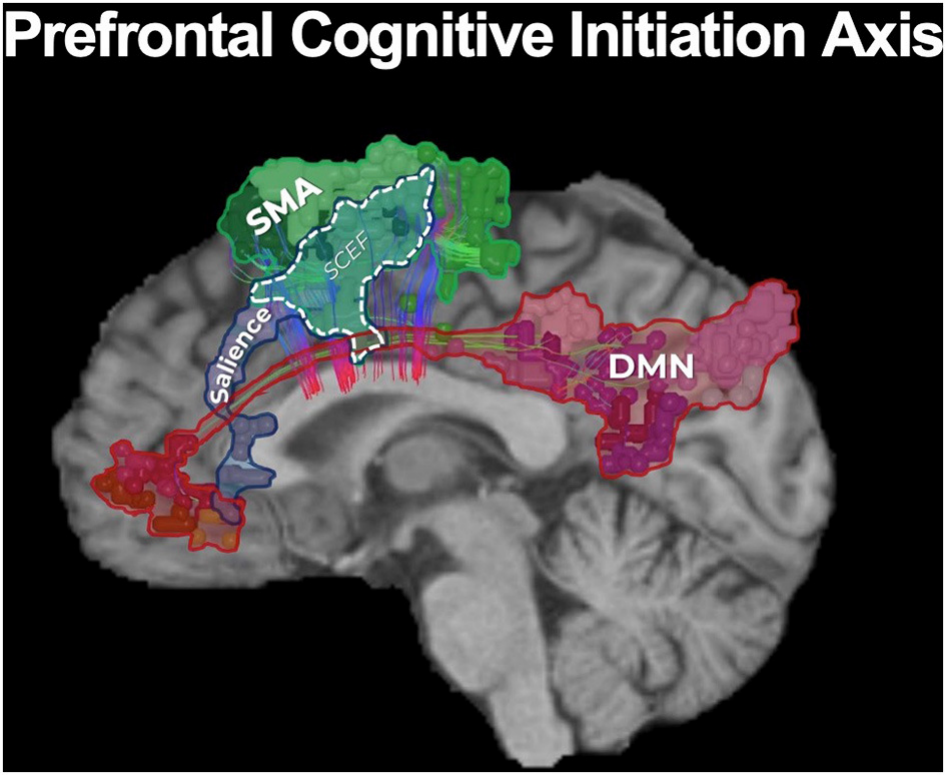
The prefrontal cognitive initiation “axis.” Our model of the initiation axis suggests that the DMN, connected via the cingulum, and the SN, connected via the frontal aslant tract (FAT), create a structural chain that extends up to the SMA in the medial frontal lobe (77). In fact, the SMA and salience networks share a node known as SCEF (supplementary and cingulate eye field). The identity and functional relevance of these connections have been supported by multiple lines of evidence (85), together suggesting this initiation “axis” is likely responsible for internally modeling goal initiation, such as with the initiation of speech and motor planning. Unsurprisingly, disruption of the integrity of this “axis” when operating on a tumor in the medial frontal lobe can lead to akinetic mutism and abulia (14, 82, 85).

The functional anatomy of the frontal lobe has long been poorly understood, with previous models vaguely suggesting that bifrontal injury causes akinetic mutism and abulia without actually providing a guide on how to avoid such deficits (84). These deficits can generally be thought of as difficulties with the initiation of internally motivated actions, presenting as a lack of self-initiated activity. Fortunately, connectomic data has improved our basis for understanding and avoiding neurologic deficits associated with tumors located along the medial frontal control networks. A model we recently proposed, in what we deemed as the prefrontal cognitive initiation “axis,” in brief suggests that the DMN, connected via the cingulum, and the SN, connected via the frontal aslant tract (FAT), create a structural chain that extends up to the SMA in the medial frontal lobe (Figure 4) (77). This initiation “axis” is likely responsible for internally modeling goal initiation, such as for the initiation of speech and motor planning, and is supported by multiple lines of evidence providing considerable insight into cognitive outcomes when operating in this area (85). For instance, anecdotal experience has previously suggested that unilateral cingulate transgression is only tolerated by some individuals, while others often develop akinesia or abulia, and that preserving the anterior cingulate can reduce such deficits. Instead, connectomic data provides more clear information on how to avoid these injuries and why they occur. Namely, the DMN contains anterior and posterior cingulate clusters linked via cingulum fibers, and disconnecting this cingulum bundle in the initiation “axis” is what likely causes abulia (73, 86). By applying this tractographic information when operating on anterior butterfly gliomas, a cingulum sparing technique can nearly completely prevent akinesis and abulia compared to not sparing the cingulum fibers (Figure 1) (82).

Furthermore, a well known post-operative neurologic deficit when operating in the medial frontal lobe is SMA syndrome, characterized by transient hemiplegia and mutism. Previous localizationist views have suggested that this deficit occurs primarily due to surgical insult in the posteromedial bank of the superior frontal gyrus, yet other patients too demonstrate varying degrees of mutism and hemiplegia when operating outside this canonical SMA (14). Fortunately, further insight on the major connectivity of the medial frontal lobe has revealed that the FAT is the principle pathway linking the SMA to premotor areas and area 44 (Broca's area) and also links hubs of the salience network, supporting a possible role of the FAT in initiating motor or speech activity through its interconnections throughout the initiation axis (87, 88). We have recently shown that by applying this knowledge in patients with gliomas in the SMA, intraoperative identification and subsequent preservation of the FAT can reduce SMA syndrome compared to just “avoiding the posterior bank of the SFG” (Figure 2). Thus, connectomic data can provide us helpful information to make more informed decisions during cerebral surgery regarding how to actually avoid causing specific neurologic deficits when considering the underlying network connectivity relative to tumor topography.

### Toward “Disconnection Surgery”

**Figure 5:**
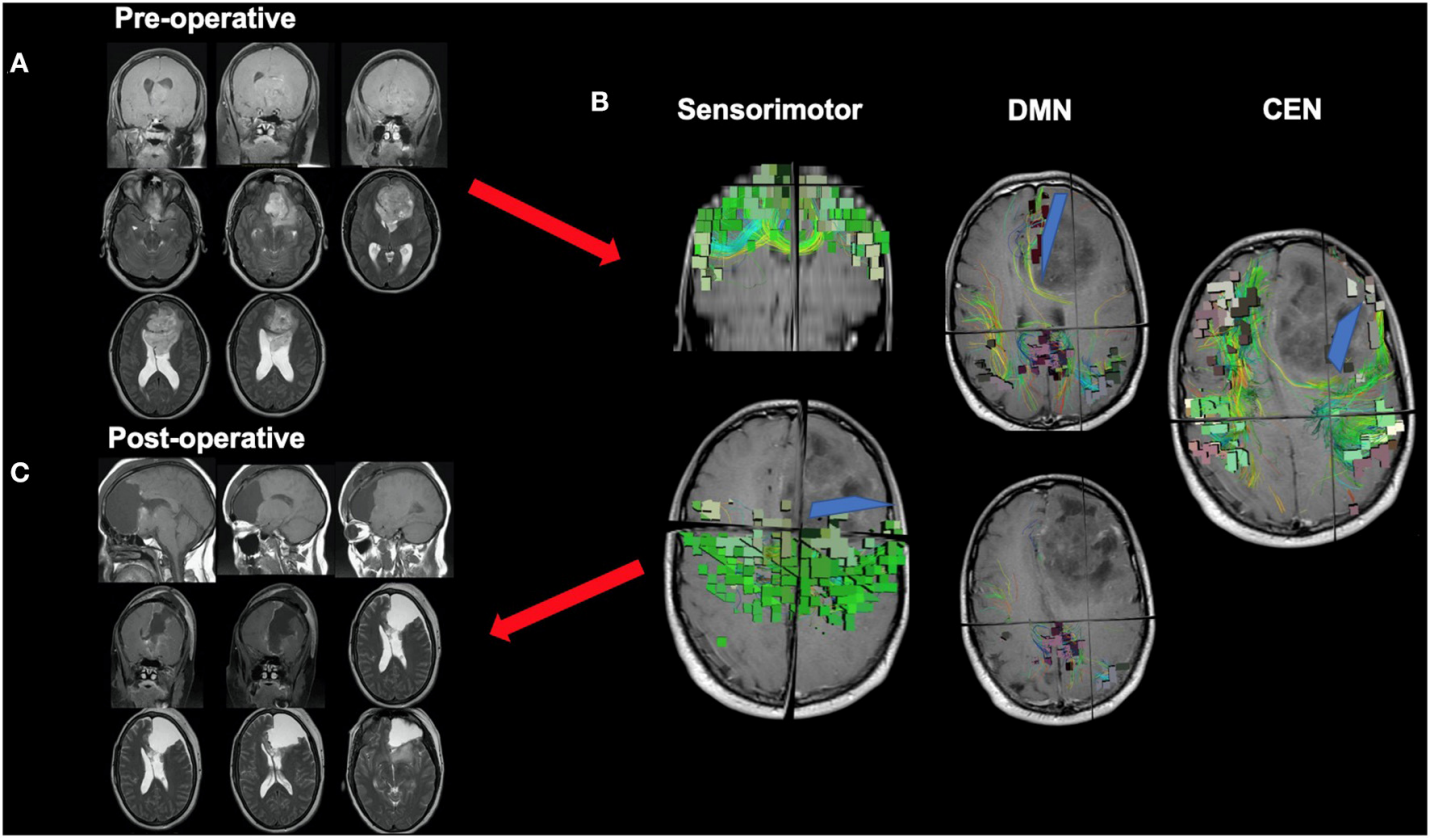
Disconnection surgery. The neurosurgical community should move toward thinking of tumor surgery as a series of cortical and subcortical disconnections to minimize unnecessary network disturbances based on tumor location and patient goals. This figure presents a case of a medial anterior, left frontal glioma which demonstrates complex relationships with adjacent anatomy, such as the corpus collosum and falx cerebri. **(A)** While a complex case, it can be reduced to a series of disconnections that can be defined against three known brain networks: the DMN on medial boundary, the CEN on the lateral boundary, and the sensorimotor network on the posterior boundary. **(B)** Understanding information on the spatial relationship of the tumor to relevant white matter tracts and major networks can allow the neurosurgeon to make more informed decisions during surgery, such as where or how far to disconnect (blue lines) normal tissue infiltrated by a tumor up until certain key fibers or nodes are met. This decision should be made based on patient pre-defined goals and patient prognosis among other factors, and further work will hopefully clarify how much of specific networks can be safely disconnected without compromising certain functions **(C)**.

Ultimately, it may be best if we begin to move away from the idea of “surgical resections” and instead toward the idea of oncological “disconnection surgery.” Such a framework contextualizes tumor surgery as a series of cortical and subcortical disconnections to minimize unnecessary multi-network disturbances based on tumor location and pre-defined patient goals. In addition to the consequences observed when disrupting the cores of the main cognitive networks as described above, it is likely that similar damage and associated consequences can be scaled to the more subtle networks if their network architecture around a tumor is not considered. Figure 5 demonstrates a case of a medial anterior, left frontal glioma. One can see that it is a tumor of substantial size, but it also involves complex relationships given it can be seen herniating under the falx cerebri, but also crossing the corpus collosum and also demonstrating an intraventricular portion. It is very easy to think of this as a “ball” of tumor, but in reality it demonstrates important relationships to large scale brain networks. Therefore, instead of thinking of the surgery as a resection, this case can be thought of as a series of disconnections that can be defined against three known brain networks to maximize the extent of resection, to minimize impairments in defined cognitive functions, and to meet patient onco-functional goals (89).

While we suggest preserving the core of the main cognitive networks is of the utmost importance, it must also be known that it may not be surgically feasible nor practical to preserve these networks depending on the tumor location and patient prognosis, a dynamic which inherently represents the concept of optimizing the onco-functional balance. While we may choose to ignore the surrounding networks and focus on the tumor alone, connectomic data still provides valuable data that informs our actual decisions during surgery and the substantial risks associated with certain tumors. Previously, decisions on cognitive preservation during cerebral surgery were being made with incomplete information. Within this concept, future improvements will hopefully clarify our ability to quantitatively measure and understand exactly how much of what specific brain networks can be disconnected without compromising the multi-network communication necessary for effective human functioning.

## Consider The Full Brain Ramifications of The Action

### Essentiality and Redundancy in Intracerebral Neurosurgery

Preservation of essential neurocognitive functions that allow for activities of daily living distills into two fundamental principles—essentiality and redundancy. A tremendous amount of time and resources are devoted to preoperative discussions with patients, as are intraoperative maneuvers regarding the need for different types of physiological monitoring. Yet, seldom do we truly challenge what eloquent tissue is worth saving and what we cannot save in given situations. Essentiality, paradoxically may not matter if a patient is already paralyzed from a tumor that has completely infiltrated the motor strip.

**Figure 6:**
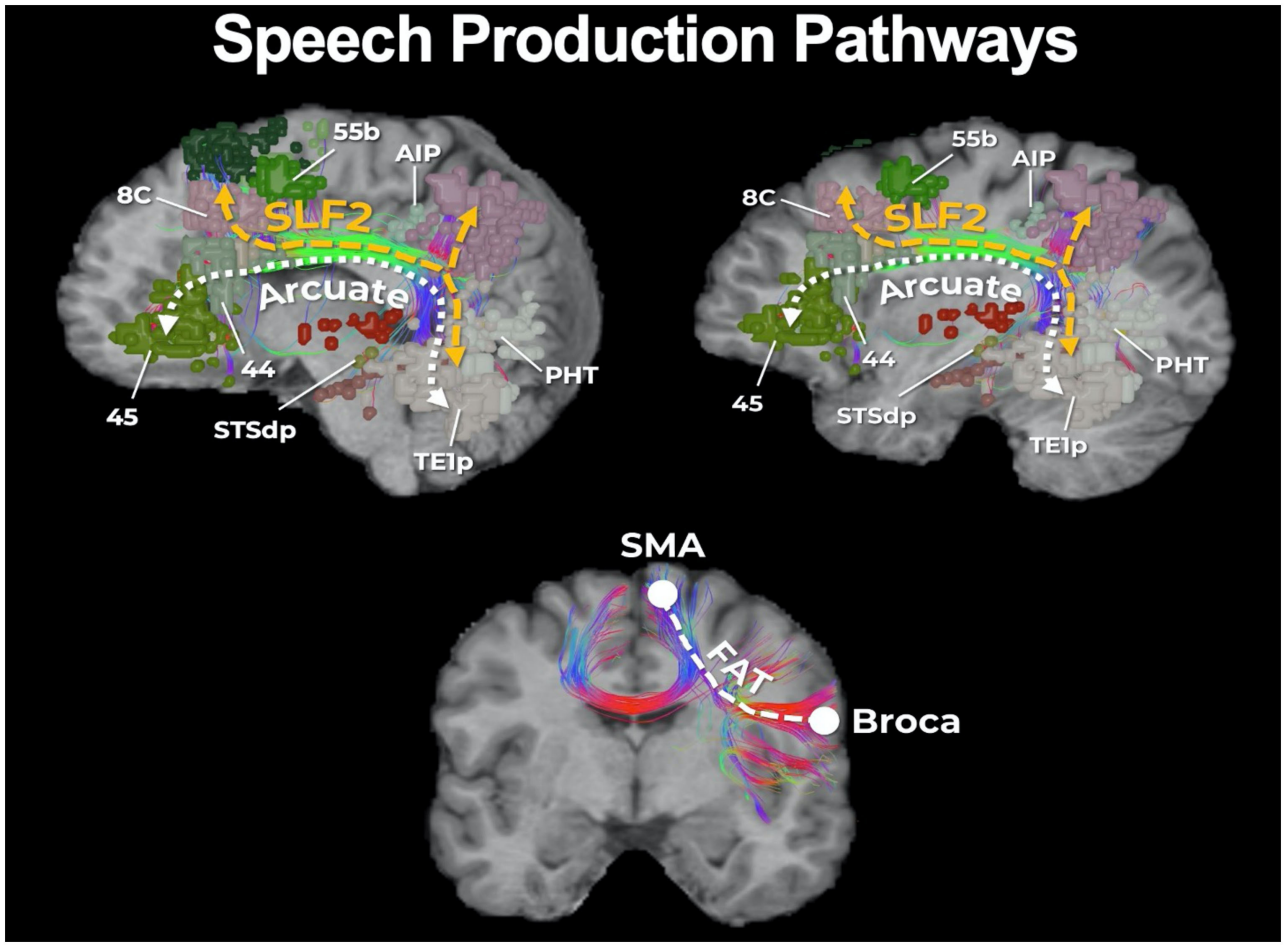
Core parcellations and fibers involved in speech production. It is important to be aware of the core parcellations involved in speech production, as well as the main fibers interconnecting those regions such as the second layer of the superior longitudinal fasciculus (SLF-II), the arcuate fasciculus (AF), and the frontal aslant tract (FAT). For instance, area 55b is a recently discovered region that increasing evidence has suggested is necessary for phonation and motor control of the larynx (90). 55b is connected to the posterior temporal language areas via the SLF-II in the left hemisphere. If unaware of such connections, transgressing 55b leads to apraxia of speech (91).

The notion of *essentiality* in supratentorial, intracerebral surgeries is based on functions that are needed to maintain a degree of quality of life. A prototypical example of this would be maintaining a functional language network. Language is deemed essential as without it one cannot interpret what is spoken to them or express their wishes, and subsequently interactions with others and the outside world would become limited. In order to preserve language processing and speech functions, the neurosurgeon should not focus on one single anatomical region but rather focus on a network, consisting of high nodal connectivity. Specifically from the inferior frontal gyrus, to the inferior parietal lobule, to the posterior temporal lobe and the fiber tracts (superior longitudinal fasciculus and arcuate fasciculus) that interconnect those anatomical domains (Figure 6). The concept of eloquent brain ought to be defined not as a single anatomical region, but to anatomical locations and their interconnected networks and damage should be interpreted accordingly (92, 93). However, an important point we want to bring out is that currently, there are no adequate models or tools that allow us to predict or even visualize “essentiality” or its related networks prior to surgery. Furthermore, as detailed earlier, it is currently unknown the degree to which specific fibers and how much of those specific fibers in such pathways can be safely disconnected without comprising the entire function of a network. We discuss this further in later sections as it remains an important avenue of future research.

When dealing with *redundancy* in neurocognitive functions, we have to take into consideration the laterality of a network and whether there are compensatory mechanisms should an area of the brain be disconnected. A prime example of this is the supplementary motor area as part of the somatosensory network. As previously mentioned, it is well-recognized that disconnection of the core of the SMA can result in hemiparesis, but the deficit is often transient in nature (94). One possibility offered through connectomic data is that compensation may occur if transcallosal projections of the FAT fibers (“crossed FAT”) are preserved, given their ability to maintain interhemispheric connectivity by connecting the contralateral SMA and premotor area with the ipsilateral motor network (82). This phenomenon is likely dependent on already existing crossing fibers as neuronal regeneration is limited in the adult population (95). In this regard, there is known redundancy in the SMA area that can further guide our surgical decisions, such as to preserve these crossed FAT fibers if possible, and can also inform us about mechanisms of patient recovery post-operatively through specific pathways. In the same manner, we often perform complete right frontal lobectomies especially in light of supramaximal resections. Although the right frontal lobe can harbor important networks, including the DMN and CEN, we know that most patients can go on to live otherwise normal lives. There remains a paucity information in understanding whether a portion of brain can be sacrificed safely due to redundancy in functions.

### Hubness Is the New Eloquence

Brain eloquence was traditionally defined as a region that houses a known neurological function, and if injured, results in a disabling neurological deficit (96–98). *How then do we define eloquent brain when planning tumor resection in a connectomics framework*? By utilizing pagerank centrality in graph theory, Ahsan et al. demonstrated that eloquent brain areas can be defined as highly connected brain hubs (93). It turns out that traditional eloquent areas of the brain are regions of high nodal connectedness. When we view the brain as an organ woven together by various networks in a mathematical manner, these hubs coincide with anatomic regions that were described to have high surgical importance by Spetzler and Martin (93, 98). Since graph theory analysis is not limited by physical distortion of the anatomy by mass-occupying pathologies, it may allow for the prediction of eloquence more accurately than anatomical landmarks. This is important given our traditionally better understanding of the general anatomy of regions responsible for language, motor and visual functions compared to that which is responsible for higher-order cognitive functions, like emotion.

Importantly, inter-individual differences exist on a macroscopic brain level in white matter connections as well as all the way down to the genetic makeup of individual cells (99). Fortunately, unique patient hubs can be determined by graph theory utilizing individual neuroimaging data in comparison to group-calculated averages, or probabilistic atlases. Areas of unexpected importance, or unexpected hubs, can be as high as 40% of all parcellations that are independent of gross anatomy (100). This is important given that outdated brain atlases based on group-averaged data or single subjects may overlook hubs unique to specific individuals, such as in the temporal pole which is often considered non-eloquent and therefore “safe to resect” (101). Recent improvements in connectomic data has also allowed others to expand the clinical utility of DES in identifying critical hubs on an individualized basis (89, 102). However, similar to graph-theory based analyses, further work is necessary to clarify how complex stimulation effects can allow us to draw stronger and more reliable conclusions about critical brain network functioning for higher-order cognition.

### Computational Measures of Cognition and Surgery Can Guide Clinical Decisions

As previously mentioned, localizationists often propose that damage to a single area provides the basis underlying loss of higher-order cognitive functions, whether induced by surgery or by the lesion. However, as described above, connectomic or network-based approaches can instead provide us more accurate models describing this pathophysiology given that a cognitive impairment is often more accurately related to the disconnection of large fiber bundles connecting multiple regions in a network (103). Unsurprisingly, intelligence (i.e., fluid intelligence) does not localize to a single area, but rather involves a series of cortical regions maintained in and interacting between their networks (104). An example of a dynamic, multi-network interaction underlying higher-order cognitive functioning can be seen with complex mathematical thinking. Utilizing meta-analytic software to aggregate task-based fMRI data in the literature concerning mathematical operations, one would identify that mathematical skills implicate a variety of brain regions across visual, semantic, motor, and DAN networks as well as the white matter connections between them. Therefore, in neurosurgery, we must consider the use of advanced computational algorithms to predict how our surgical cuts affect *multiple networks* that function together to facilitate higher-order functions if we are to optimize cognitive morbidity following surgery.

Graph-based network analyses may allow us to better consider these multi-network interactions by measuring the possible effects of lesions or surgical disconnections on general cognitive functioning. *Global efficiency* is one example and it is defined as the average inverse of the shortest distance between two nodes in a brain network, producing a value that represents the capacity for information transfer on a global level (105). The length of a path represents the potential routes of information flow in the brain and therefore it is often considered that the shorter the path, the stronger the potential for functional integration (106). What is particularly advantageous of such data-driven approaches is that the data can be non-invasively amalgamated from fMRI and DTI techniques. Therefore, patient neuroimaging data can be easily input into these complex algorithms to produce simple, interpretable scores for neurosurgeons to examine (i.e., “a higher global efficiency is better”).

Additionally, these analyses provide a safe platform for further surgical decision making as there are computational methods to analyze how resilient an individualized brain network is to a particular insult in the perioperative period. For instance, percolation theory attempts to estimate the minimal set of essential nodes in a brain network to effectively transfer information (106). Therefore, simulated lesions or removal of nodes (*simulated brain surgery*) can be safely performed on patient structural connectivity graphs before or after surgery and then analyzed with measures of global efficiency to understand how a patient might be affected by a specific surgical decision or to understand beneficial avenues for subsequent neurorehabilitation (107). Also, as many neurologic disorders are neurodegenerative occurring throughout a long-term period, such computational analyses can also be applied to gauge a patient's cognitive functioning over time to guide future planning of care. While our team is actively utilizing similar methodology, there is a dearth of research which has yet to link these computational models with clinical outcomes. Given that differences in methodology concerning relatively nascent big data approaches may produce heterogenous results, especially on an individual patient basis, further work must be done to investigate the clinical relevance of such computational models.

## Move Our Thinking Toward Individual Circuits

### The Transdiagnostic Hypothesis

*Are there areas in individual human brains that if resected during tumor surgery, can lead to symptoms of anxiety or depression?* Such a question must be considered given the severity of post-operative cognitive morbidity which is possible, similar to the observation mentioned above that individual brains may display unique hub areas that if cut can result in unexpected dramatic losses of cognitive functioning (100). However, to answer a complex question such as this, we must strive to get down to the level of individual connectomes and neural microcircuits, and this requires big data that can be best handled with advanced computational algorithms offered with machine learning (ML) (108). In a cohort of patients with Alzheimer's disease who underwent rsfMRI and diffusion tensor imaging, we utilized ML to generate and detect individual-level anomalies in the structural and functional connectome (80). As expected, some similarities were found between patients, namely that there was consistent structural white matter loss focused around the DMN and subcortical structures. However, there was also significant heterogeneity in abnormal functional connectivity between patients, suggesting the opportunity to individualize future therapeutic strategies possibly based on different clinical phenotypes, moving us steps closer to true precision medicine (80). Similar work has been presented by others in a number of disorders with different methodologies (109, 110), however further work is necessary for us to effectively understand and identify abnormal connectivity patterns between different individuals that that relate to specific symptoms.

**Figure 7:**
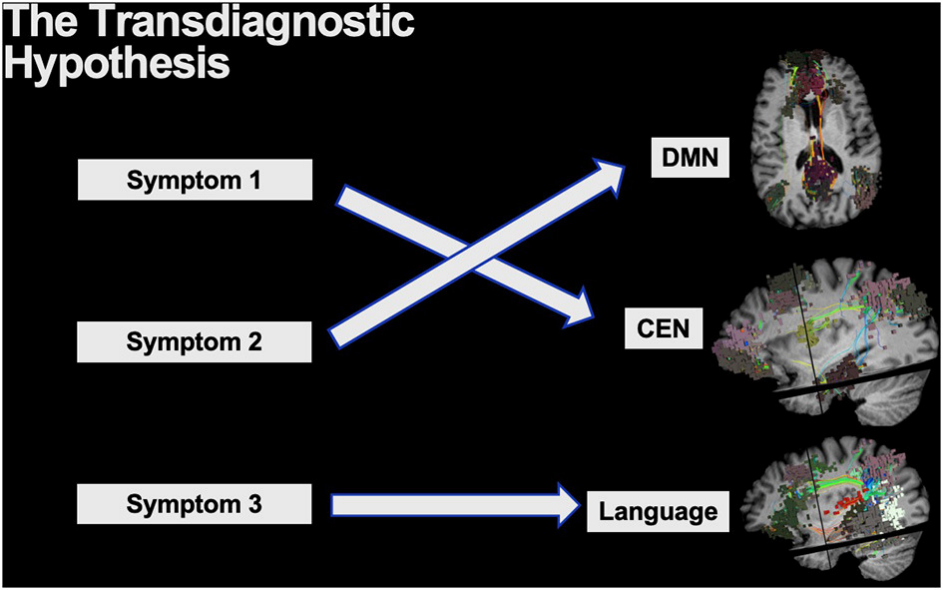
The transdiagnostic hypothesis is often applied to explain the core psychopathological symptoms across a range of psychiatric disorders. Thus, while certain disorders may be clinically grouped together with vague classifications, unique symptoms likely localize to unique brain networks providing the need for addressing them individually.

The *transdiagnostic hypothesis* is often used in psychiatry to describe the core psychopathological symptoms which underlie a range of clinical disorders (18, 111). In the same context, transdiagnostic models can cover both commonalities and differences between clinical disorders or disorder subtypes as it knocks down previous rigid barriers set by vague clinical classifications, such as by the Diagnostic and Statistical Manual of Mental Disorders (DSM). If multiple unique symptoms arise, each can be thought to generally localize to a different brain circuit requiring unique attention despite their rigid DSM clinical classifications (Figure 7). For instance, many treatments for major depression disorder (MDD) target the dorsolateral prefrontal cortex (DLPFC) for modulation, yet the responses are highly variable and not all symptoms tend to become resolved (112). While it is known that different disease-subtypes of depression exist which localize to different networks, even the common symptoms across numerous depression subtypes, such as anxiety and anhedonia, also localize to different brain networks and respond to different target selections (18). To add further complexity, while the DLPFC includes core regions of the CEN, it is a functionally heterogenous region that has been characterized into 13 distinct functional regions by the HCP that must all be precisely considered on a patient by patient basis (15). Unsurprisingly, similar complexity can also be seen in other clinically relevant regions such as the precuneus and posterior cingulate cortex which house nodes of the DMN (15, 75). Therefore, to truly understand the connectome of patients presenting with a broad range of symptoms and more effectively target and address their individual symptoms, we must strive to get to the level of microcircuits that are implicated on an individual patient basis.

### Incorporating the RDOC Framework into Future Research

If we reimagine surgical anatomy in terms of networks and advance the field with the goal of preserving various domains of neurocognition, it is important to apply a framework as a community in order to identify psychopathology-relevant constructs from the experimental literature. The Research Domain Criteria (RDoC) project was proposed by the US National Institute of Mental Health (NIMH) to classify mental illnesses that are based on dimensions of observable behavioral and neurobiological measures (113). It shifts the focus from clinical descriptions to more squarely examine aberrant mechanisms with the goal of how these mechanistic anomalies drive psychiatric symptoms. It assumes that mental disorders can be explained in terms of brain circuits and that abnormalities of these circuits are identifiable (114).

This tool from the psychiatry world can be leveraged to study neurocognitive changes after craniotomies as the literature in this regard is mostly based on the description of clinical symptoms, such as the DSM criteria for psychiatric illnesses. The application of the RDoC framework will differ in the neurosurgical field in that we can negate more developmental trajectories and environmental influences. The system includes several broad functional domains, such as negative valence, positive valence, cognitive systems, systems for social processes, arousal/modulatory systems, and sensorimotor systems (113). Each relevant domain is studied through queries of genes to circuits to behavior or self-reports, in order to provide an integrative understanding of its functioning (113). In other words, rather than merely reporting neurocognitive outcomes after awake craniotomies, one way to advance the field of research is to investigate changes in a patient's functional connectome and identify how those changes may contribute to and provide an explanation for neurocognitive manifestations after intracerebral surgeries. While these observations suggest a promising future in neuroscience with clinical applicability, there is still currently much to be done and further robust clinical evaluations are necessary to understand how we can better study and treat individualized patient connectomes (115).

## The Possibility That We Can Change The Connectome

### The Ability for Neural and Network Plasticity

Tumor resection inherently causes harm in some patients due to the locations of their pathology. If the human brain is composed of networks and each network is composed of various nodes, the question remains*: is there enough plasticity and redundancy in global cerebral networks that abnormal connections can be augmented to reduce neurocognitive or physical deficits*? The answer to this question is a resounding “yes,” but with caveats.

The brain can demonstrate a high capacity for cerebral plasticity following a number of cortical and subcortical insults. Although, most of our current understanding of cerebral plasticity comes from stroke patients and less so in glioma patients (116). Following cerebral infarction, primary motor and secondary motor cortices demonstrate functional reorganization to facilitate improved motor functioning (117). However, some computational models argue the capacity for neuroplasticity is dependent on the type of cortical damage which has occurred, especially considering temporal factors (26). For instance, acute brain damage often causes more localized neuronal cell death and may demonstrate less capacity for cerebral plasticity compared to slow growing lesions (i.e., low-grade gliomas), which often disrupt more cerebral cortex and affects more networks, but also provides longer opportunities for functional reorganization due to less abrupt neuronal death. Therefore, it seems unsurprising that patients with slow-growing lesions may demonstrate more capacity for plasticity when targeting their functional connectome compared to patients suffering from acute strokes (26). As such, others have recently extensively detailed possible atlases of neuroplastic potential for diffuse LGGs based on their connectivity (118–120).

*However, what about faster growing lesions such as in glioblastoma (GBM)?* It is important to note that there exists pathophysiological differences in strokes and gliomas which may explain the ability for cortical reorganization following *acutely* growing GBM lesions in specific instances (121). In a patient presenting with a right frontal GBM with a decreased state of consciousness despite neurosurgical intervention, rsfMRI data demonstrated an absence of the DMN. Remarkably, after five sessions of navigated intermittent theta burst stimulation as an off-label TMS therapy to try to stimulate DMN activity, the patient demonstrated drastic improvements in cognition and alertness with a partial restoration of the DMN in just 2 weeks (121). There exists a number of limitations in this example being a single case. However, this case indeed demonstrates that there is a possibility to actively promote neurorehabilitation with brain stimulation targeted at the DMN in high-grade gliomas as well despite most discussion focusing on LGGs. Individualized connectomic approaches if implemented before any modulatory treatment can identify any network reorganization occurring from the lesion and then these data can subsequently be utilized for effective connectome-based target selection to strengthen previously silent polysynaptic cortical pathways (19). As we improve our understanding of the capacity for neural plasticity in a variety of different brain lesions, we must further link these findings to the structural and functional connectome. Such work will further clarify the opportunities and also limitations in addressing possible network disruptions occurring during the perioperative period.

### rTMS for Neurorehabilitation and Restoring Onco-Functional Balance

Repetitive transcranial magnetic stimulation (rTMS) is a form of non-invasive brain stimulation that applies repeated magnetic pulses extracranially to generate an electrical current in the cortex. This in turn provokes electrophysiological changes in the target area and correlated brain networks (122). Given that a cortical insult may disrupt the oscillatory synchrony of the network that the damaged region belongs to, rTMS provides a feasible and safe way to attempt to re-establish this synchronization and reform the network. Currently, it is an evidence-based treatment mainly used for pharmacoresistant major depressive disorder (MDD), which is thought to be a disorder based on the interplay between the DMN and CEN (123). Repetitive TMS targeting parts of the DLPFC has been shown using rsMRI to selectively modulate functional connectivity both within and between the CEN and DMN (123). In the same manner, rTMS to the primary motor cortex can enhance motor performance by inducing rapid changes in the sensorimotor networks along with activation in the bilateral basal ganglia, left superior frontal gyrus, bilateral pre-SMA, right medial temporal lobe, right inferior parietal lobe, and right cerebellar hemisphere (124). Much of our previous understanding of the benefits of rTMS comes from stroke patients as well (125); however, with concepts of connectomic-based targeting in neuropsychiatric illnesses, more work is needed to be able to apply population averaged connectivity measurements on an individual level.

Importantly, as reiterated throughout the current manuscript, different patients can present with different disease subtypes demonstrating unique clinical symptoms localizing to different brain networks. Despite these complex relationships, fortunately, individualized connectomic-based TMS target selection is possible and provides an important area of future research which has been previously barren. Fox et al. illustrated the feasibility of single-subject, connectivity-guided TMS targeting within regions of the left DLPFC in two patients with depression (19). Furthermore, our team has demonstrated the feasibility of an agile, data-driven, connectomic approach for TMS target selections at the single-subject level based on rsfMRI data for generalized anxiety disorder (GAD). Still, such data-driven, connectomic approaches for rTMS and other modulatory treatments are relatively nascent in the context of individualized treatments. While already demonstrating their safety profile, findings from these studies should be refined in the future to improve and expand their clinical applicability on a larger scale ultimately assessing its reproducibility.

### Movements Forward in Connectivity-Based Modulatory Treatments

Examples above for connectome-based neurorehabilitation are mostly focused on rTMS to restore network synchrony; however, similar applications and results have been demonstrated with other modulatory treatments as well. A common method of modulating brain activity is with deep brain stimulation (DBS) of subcortical structures. Unsurprisingly, the most effective DBS subcortical targets demonstrate strong connectivity to the most effective TMS cortical targets for a number of disorders (126). For instance, while parts of the DLPFC are a favorite TMS target to treat symptoms of depression, the most effective subcortical DBS targets for depression resides in the subcallosal cingulate cortex (SCC). Connectomic data suggests these cortical and subcortical targets are strongly connected to each other and both work via modulating the same networks (127). In fact, TMS targets that demonstrate stronger connections to the SCC may demonstrate better clinical improvements in MDD (128); however, the degree to which these relationships expand to other disorders is still uncertain, but likely.

Importantly, similar limitations exist in both modalities in that their current utilization is also hampered by the lack of understanding of brain networks in different disease states and the limited validation of these connectomic-based treatments in individual patients with specific clinical phenotypes. It would be logical to select a node that serves as an important modulatory hub for altering the state of a network. However, what remains to be elucidated is target identification in many of these clinical disorders, and especially in rTMS, if the stimulation should be excitatory or inhibitory. Further, it has to be taken into account that if a crucial fiber tract (i.e., the entire corticospinal tract) is destroyed, no amount of neuromodulation will likely be able to salvage that function. Repetitive TMS, among other modulatory treatments, hold promise for neurorehabilitation, but their application will first require further improvements in our understanding of network disruptions following intracerebral surgeries.

## Conclusion

Neurocognitive decline is common after intracerebral surgeries. Outside the context of language and motor skills, the mechanisms underlying declines in various neurological domains are not well-studied likely due to our previous lack of understanding of the complete structural and functional brain connectome. It is prudent for neurosurgeons to reimagine the brain as a confluence of networks, rather than an organ comprised of isolated regions dedicated or not dedicated to specialized functions. Brain connectomics provide the neurosurgeon further information on the relationships between tumor, neuroanatomy, and cognitive functions which can be leveraged to maximize our perioperative decisions while minimizing neurocognitive declines following intracerebral surgeries. Furthermore, such connectomic-based decisions provide novel opportunities to optimize post-operative neurorehabilitation via network augmentation, and advance the field using a common research framework that can be refined over time. However, as we appropriately move toward ideas such as “disconnection surgery” and connectome-based neurorehabilitation, further work must be done together in the neuroscience and neurosurgical communities.

## Author Contributions

ND, BB, and JY: writing—original draft and writing—review and editing. MS: conceptualization, methodology, and supervision. All authors contributed to the article and approved the submitted version.

## Conflict of Interest

MS is the Chief Medical Officer of Omniscient Neurotechnology, however this does not pose a conflict of interest in this study. The remaining authors declare that the research was conducted in the absence of any commercial or financial relationships that could be construed as a potential conflict of interest.

## Publisher's Note

All claims expressed in this article are solely those of the authors and do not necessarily represent those of their affiliated organizations, or those of the publisher, the editors and the reviewers. Any product that may be evaluated in this article, or claim that may be made by its manufacturer, is not guaranteed or endorsed by the publisher.
